# Complete life cycle of a pennellid *Peniculus minuticaudae* Shiino, 1956 (Copepoda: Siphonostomatoida) infecting cultured threadsail filefish, *Stephanolepis cirrhifer*

**DOI:** 10.1051/parasite/2013041

**Published:** 2013-10-29

**Authors:** Norshida Ismail, Susumu Ohtsuka, Balu Alagar Venmathi Maran, Satoshi Tasumi, Kassim Zaleha, Hirofumi Yamashita

**Affiliations:** 1 Takehara Marine Science Station, Graduate School of Biosphere Science, Hiroshima University, 5-8-1 Minato-machi Takehara, Hiroshima 725-0024 Japan; 2 Faculty of Agriculture, Biotechnology and Food Science, University Sultan Zainal Abidin, Kampus Gong Badak 21300 Kuala Terengganu, Terengganu Malaysia; 3 Marine Ecosystem Research Division, Korea Institute of Ocean Science & Technology P.O. Box 29 Ansan, Seoul 425-600 Korea; 4 Fisheries Laboratory, Graduate School of Agricultural and Life Sciences, The University of Tokyo, 2971-4 Bentenjima, Maisaka, Nishi-ku, Hamamatsu Shizuoka 431-0214 Japan; 5 Fisheries Department, Faculty of Fisheries and Aqua-Industry, University Malaysia Terengganu 21030 Mengabang Telipot, Kuala Terengganu Terengganu Malaysia; 6 Ehime Prefecture Aquaculture Research Group Promotion Office of Agriculture, Forestry and Fisheries Research Institute, Fisheries Research Center 5516 Shita-ha Uwajima Ehime Prefecture 798-0104 Japan

**Keywords:** Copepoda, Pennellidae, Development, *Peniculus minuticaudae*, Threadsail filefish, Life cycle

## Abstract

The complete life cycle of a pennellid copepod *Peniculus minuticaudae* Shiino, 1956 is proposed based on the discovery of all post-embryonic stages together with the post-metamorphic adult females infecting the fins of threadsail filefish *Stephanolepis cirrhifer* (Monacanthidae) cultured in a fish farm at Ehime Prefecture, Japan. The hatching stage was the infective copepodid. The life cycle of *P. minuticaudae* consists of six stages separated by moults: the copepodid, four chalimi and adult. In this study, the adult males were observed frequently in precopulatory amplexus with various stages of females however, copulation occurs only between adults. Fertilized pre-metamorphic adult females carrying spermatophores may detach from the host and settle again before undergoing massive differential growth into the post-metamorphic adult female. Comparison of the life cycle of *P. minuticaudae* has been made with three known pennellids: *Lernaeocera branchialis* (Linnaeus, 1767), *Cardiodectes medusaeus* (Wilson, 1908) and *Lernaeenicus sprattae* (Sowerby, 1806). Among the compared species, *P. minuticaudae* is the first ectoparasitic pennellid to be discovered to complete its life cycle on a single host without any change in infection site preferences between infective copepodid and fertilized pre-metamorphic female.

## Abstract (fr)

Le cycle de vie complet d’un copépode Pennellidae *Peniculus minuticaudae* Shiino, 1956 est proposé sur la base de la découverte de tous les stades post-embryonnaires et des femelles adultes post-métamorphiques infectant les nageoires du poisson Monacanthidae *Stephanolepis cirrhifer* cultivé dans une ferme piscicole de la préfecture d’Ehime, Japon. Le stade d’éclosion est le copépodite infectieux. Le cycle de vie de *P. minuticaudae* se compose de six stades séparés par des mues : le copépodite, quatre chalimi et les adultes. Dans cette étude, les mâles adultes ont été fréquemment observés en amplexus précopulatoire avec différents stades femelles mais l’accouplement a lieu seulement entre adultes. Les femelles adultes pré-métamorphiques fécondées transportant des spermatophores peuvent se détacher de l’hôte et s’installer à nouveau avant de subir une croissance différentielle massive en femelles adultes post-métamorphiques. La comparaison du cycle de vie de *P. minuticaudae* a été faite avec trois Pennellidae connus : *Lernaeocera branchiali*s (Linnaeus, 1767), *Cardiodectes medusaeus* (Wilson, 1908) et *Lernaeenicus sprattae* (Sowerby, 1806). Parmi les espèces comparées, *P. minuticaudae* est le premier Pennellidae ectoparasite pour lequel on a découvert que le cycle de vie peut s’effectuer sur un seul hôte sans aucun changement dans les préférences de site d’infection, depuis le copépodite infectieux jusqu’à la femelle fécondée pré-métamorphiques.

## Introduction

The genus *Peniculus* von Nordmann, 1832 (Copepoda: Siphonostomatoida: Pennellidae) consists of 14 nominal species [3, 30]. In Japan, three *Peniculus* species have so far been recorded: *P. minuticaudae* Shiino, 1956, *P. ostraciontis* Yamaguti, 1939 and *P. truncatus* Shiino, 1956 [27, 32]. *Peniculus minuticaudae* has so far been recorded from fishes of two different families: four fish hosts of the family Monacanthidae, threadsail filefish *Stephanolepis cirrhifer* (Temminck and Schlegel, 1850), black scraper *Thamnaconus modestus* (Günther, 1877), unicorn leatherjacket filefish *Aluterus monoceros* (Linnaeus, 1758), hairfinned leatherjacket *Paramonacanthus japonicus* (Tilesius, 1809) and one Chaetodontidae, brown-banded butterflyfish *Chaetodon modestus* Temminck and Schlegel, 1844 [20, 24, 27, 30]. *Peniculus ostraciontis* was recorded from two boxfishes, humpback turretfish *Tetrosomus gibbosus* (Linnaeus, 1758) and the triangular boxfish *T. concatenatus* (Bloch, 1785) (Ostraciidae) [32, 28], while *P. truncatus* was found to infect rockfish *Sebastes oblongus* Günther, 1877 [27] and Korean rockfish *S. schlegelii* Hilgendorf, 1880 (Sebastidae) [30].

Shiino [27] first described the post-metamorphic female of *P. minuticaudae* recovered from wild *S. cirrhifer* collected from the waters off Shirahama, Wakayama Prefecture, Japan. The post-metamorphic female of *P. minuticaudae* has recently been redescribed from Japanese [24] and Korean [30] waters. Recent reports indicated the severity of infestation by *P. minuticaudae* on fishes kept in captivity such as in aquaculture facilities [10, 20, 30] and in a commercial aquarium [24].

The life cycle of pennellids can be direct or indirect depending on taxon [25]. Some utilize two hosts, i.e., intermediate and definitive [7, 8, 19, 25, 29], while some utilize only one host [26]. Based on the discovery of different developmental stages (copepodid, late chalimus stages, pre-metamorphic adult female and adult male) on a host which was kept in an aquarium without any possible secondary host, it was recently suggested that *P. minuticaudae* could complete its life cycle on a single host [24]. In the present study, we found all stages including copepodid, chalimi, adults and post-metamorphic females on the fins of cultured *S. cirrhifer*, indicating that *P. minuticaudae* could complete its life cycle on a single host. We also confirmed that the hatching stage of *P. minuticaudae* is the copepodid, which is relatively rare in copepods although within the Pennellidae both types of hatching, naupliar and copepodid, are known [8, 12, 15, 25, 26, 29].

## Materials and methods

### Observation of the first hatching stage

Ovigerous post-metamorphic adult females of *P. minuticaudae* (*n* = 10) were collected from the fins of *S. cirrhifer* captured from the sea cage aquaculture facilities of the Fisheries Research Center, Ehime Research Institute of Agriculture, Uwajima, Ehime Prefecture, Japan (33°16′92″ N, 132°43′94″ E) on 21 November 2011. Egg strings were carefully detached from the ovigerous females using fine forcep then transferred into vials containing filtered sterilized seawater before being transported to Takehara Marine Science Station, Hiroshima, Japan (34°32′58″ N, 132°92′33″ E) for incubation. In the laboratory, the egg strings were transferred into Petri dishes containing fresh filtered sterilized seawater and incubated (NK System Biotron LH-200-RDSCT, Tokyo) at a temperature of ca. 22–25 °C until hatching. Hatching of copepodids was confirmed by direct observation under an Olympus SZX7 dissecting microscope. All hatching copepodids were immediately preserved in 70% ethanol for further study.

### Description of developmental stages

Twenty individuals of *S. cirrhifer* (fork length 15–21 cm) were obtained from the Fisheries Research Center on 26 June 2011 and preserved in 10% neutralized formalin seawater individually in a plastic bag. They were screened for infection with copepods especially on the fins. The preservative in each bag was also filtered through a 300 μm sieve to find any detached specimens. The collected specimens were preserved in 70% ethanol.

All stages were described except the adult male and post-metamorphic adult female since both are already well described [24, 27, 30]. Prior to making observations, specimens were cleared in a drop of lactophenol for 30 min, dissected and examined following the wooden slide procedure [13]. Drawings and measurements were made with the aid of a drawing tube attached to an Olympus BX50 differential interference contrast microscope. Measurements are given as mean (minimum-maximum). Specimens were measured intact using an ocular micrometer. Anatomical terminology follows Kabata [18] and Huys & Boxshall [14] and fish names conform to FishBase [9].

### Scanning electron microscope (SEM) analysis

Five specimens of each life stage of *P. minuticaudae* were used for scanning electron microscopy (SEM). The copepods were transferred to 70% ethanol and then dehydrated through a graded series of ethanol (90%, 99.5% and 100%) and finally in isoamyl acetate. The samples were critical point-dried using CO_2_ gas and ion-sputtered for observation with a JSM6510-LV scanning electron microscope (JEOL, Tokyo).

## Results

### Hatching of copepodid

Eight copepodids hatched from a single egg string after 27 h of incubation and were observed to move around after hatching. Some other copepodids from the same egg string hatched with a layer of membrane which hindered their movement and sank to the bottom of the Petri dish. Some were not completely released from the egg string. The active copepodids were collected and preserved in 70% ethanol for description. After three days, observations on the other egg strings were discontinued due to contamination.

### Description

#### Copepodid (Figures 1A–K, 2A–B)

Body length: (based on six individuals hatching from incubated egg string), 2.87 (2.60–3.26) mm; (based on five individuals collected from the host), 3.12 (2.99–3.22) mm.

**Figure 1.**
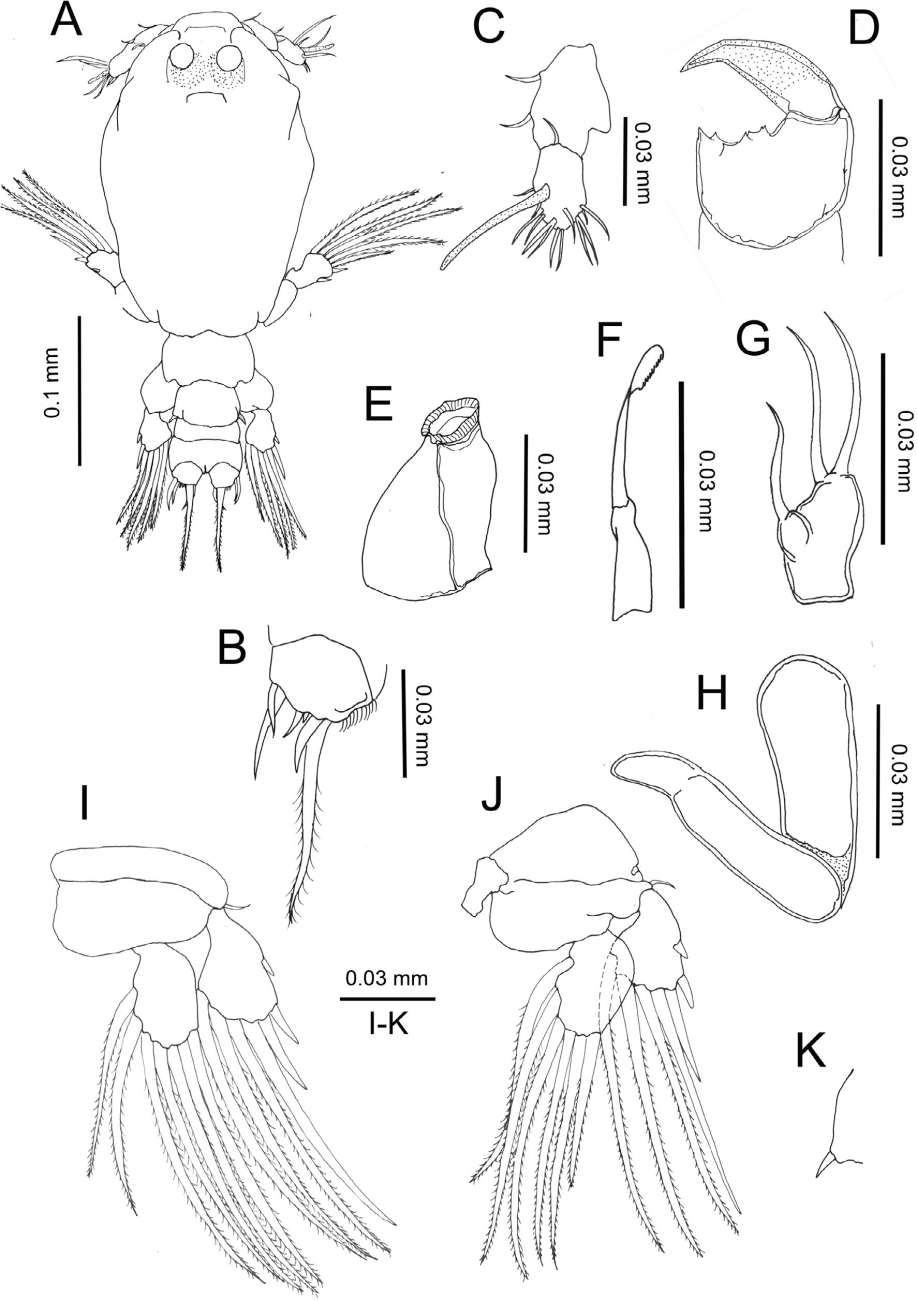
*Peniculus minuticaudae* Shiino, 1956. Copepodid stage: A, habitus, dorsal view; B, caudal ramus, dorsal view; C, antennule; D, antenna; E, oral cone; F, mandible; G, maxillule; H, maxilla; I, leg 1, anterior surface; J, leg 2, anterior surface; K, leg 3, dorsal view.

**Figure 2.**
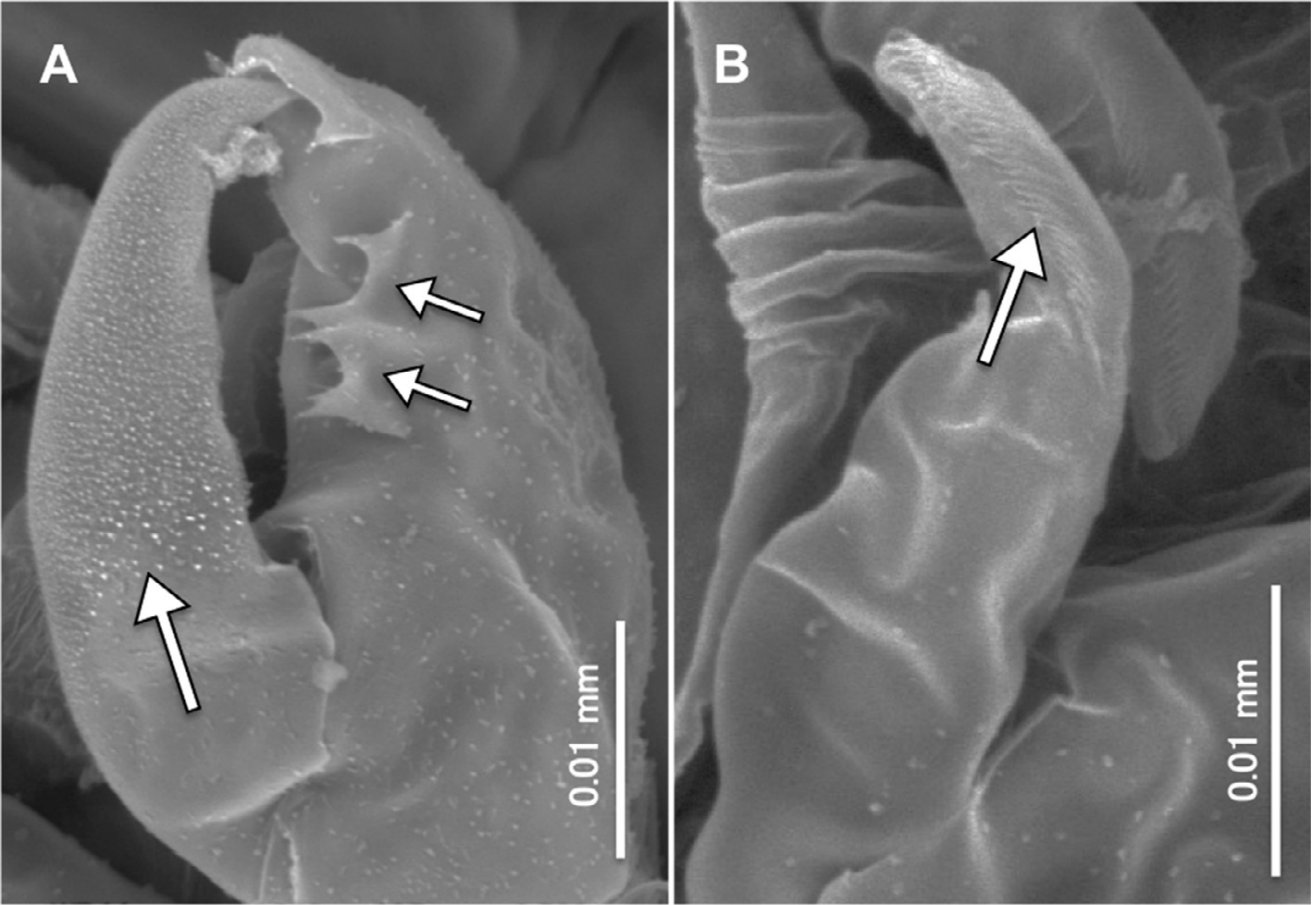
*Peniculus minuticaudae* Shiino, 1956. Copepodid stage, scanning electron micrographs: A, antenna. Arrows showing spinules on terminal claw and the tooth-like protuberances on middle segment; B, maxilla. Arrows showing transverse striations on posterior part.

Body (Figure 1A) oval with dorsal surface highly pigmented from anterior part of cephalothorax to caudal rami (pigmentation omitted from illustration). Rostrum weakly developed, rounded. Cephalothorax incorporating first pedigerous somite, about twice as long as free post-cephalothoracic somites and caudal rami combined. Widest about mid-length. Naupliar eyes conspicuous. Second pedigerous somite wider than long; third pedigerous somite with anlagen of leg 3 (Figure 1K) represented by paired, laterally-located papillae, each bearing one short spine; third free somite shorter than preceding somite, unarmed; fourth free somite bearing caudal rami (Figure 1B) armed with single long spinulose seta and five short naked setae. Inner surface of ramus ornamented with row of fine setules.

Antennule (Figure 1C) 2-segmented, proximal segment bearing 3 setae; terminal segment armed with 13 setae and aesthetasc. Antenna (Figures 1D and 2A) incompletely 3-segmented; proximal segment large; middle segment broad with two pointed processes posteriorly; two pairs of tooth-like protuberances along inner margin; terminal segment claw-like with spinules. Oral cone (Figure 1E) located on mid-ventral line, labrum and labium not fused, each forming about half of oral cone. Mandible (Figure 1F) slender, proximal part cylindrical, distal part loosely inserted into mouth cone, flat with 10 teeth at tip. Maxillule (Figure 1G) indistinctly bilobed, carrying 1 and 2 distal setae, respectively. Maxilla (Figure 1H) 2-segmented; proximal segment large, rod-like; distal segment curved, ending in blunt tip with transverse striations on posterior part (see Figure 2B). Maxilliped absent. Legs 1 (Figure 1I) and 2 (Figure 1J) with coxa, basis and unisegmented rami. Armature formula of legs shown in Table 1.

**Table 1.** Armature formula of legs of six different stages in the life cycle of *Peniculus minuticaudae* Shiino, 1956 (Roman and Arabic numerals indicating spines and setae, respectively).

Stages/Legs	Segmentation			
Copepodid	Coxa	Basis	Exopod	Endopod
Leg 1	0-0	1-0	II, II, 3	7
Leg 2	0-0	1-0	II, II, 3	6
Chalimus I	Protopod		Exopod	Endopod
Leg 1	1-0		7	7
Leg 2	1-0		7	6
Chalimus II				
Leg 1	1-1		7	7
Leg 2	1-0		8	8
Leg 3	0		2	–
Chalimus III				
Leg 1	1-1		8	8
Leg 2	1-0		9	8
Leg 3	0-0		6	–
Leg 4	0-0		5	–
Chalimus IV				
Leg 1	1-1		9	8
Leg 2	1-0		10	8
Leg 3	1-0		6	–
Leg 4	1-0		5	–
Pre-metamorphic adult female	Coxa	Basis	Exopod	Endopod
Leg 1	0-0	1-1	I-1; I, I, 5	0-1; 7
Leg 2	0-0	1-0	I-1; I, I, 6	0-1; 7
Leg 3	0-0	1-0	0-0; I, 5	–
Leg 4	0-0	1-0	0-0; I, 4	–

##### Remarks

The copepodid of *P. minuticaudae* collected from the host *P. japonicus* [24] is similar to the hatching copepodid of *P. minuticaudae* in our study except for its larger size. Through SEM examination, some features of the antenna (Figure 2A) and maxilla (Figure 2B) are given in detail. The surface of the terminal segment of the antenna is ornamented with small spinules and along the inner margin of the second segment there are two pairs of tooth-like protuberances; the innermost element is bifurcated (Figure 2A). Among pennellids, only *P. minuticaudae* shows these features, but the antennae of *Cardiodectes* sp. [12] and *Lernaeenicus sprattae* (Sowerby, 1806) [26] are similar to those of chalimi of *P. minuticaudae*. Recently, Brooker et al. [8] redescribed the copepodid of *Lernaeocera branchialis* (Linnaeus, 1767) and reported that the distal border of the antenna is ornamented with blunt processes rather than a spine. Unlike *L. branchialis* where sexual dimorphism can be detected even at the copepodid stage through the setal size (finer in female) in the caudal ramus [8], no sexual dimorphism was detected in *P. minuticaudae*.

#### First chalimus, female (Figure 3A–J)

Body length (based on five individuals collected from *S. cirrhifer*): 3.22 (3.13–3.35) mm.

**Figure 3.**
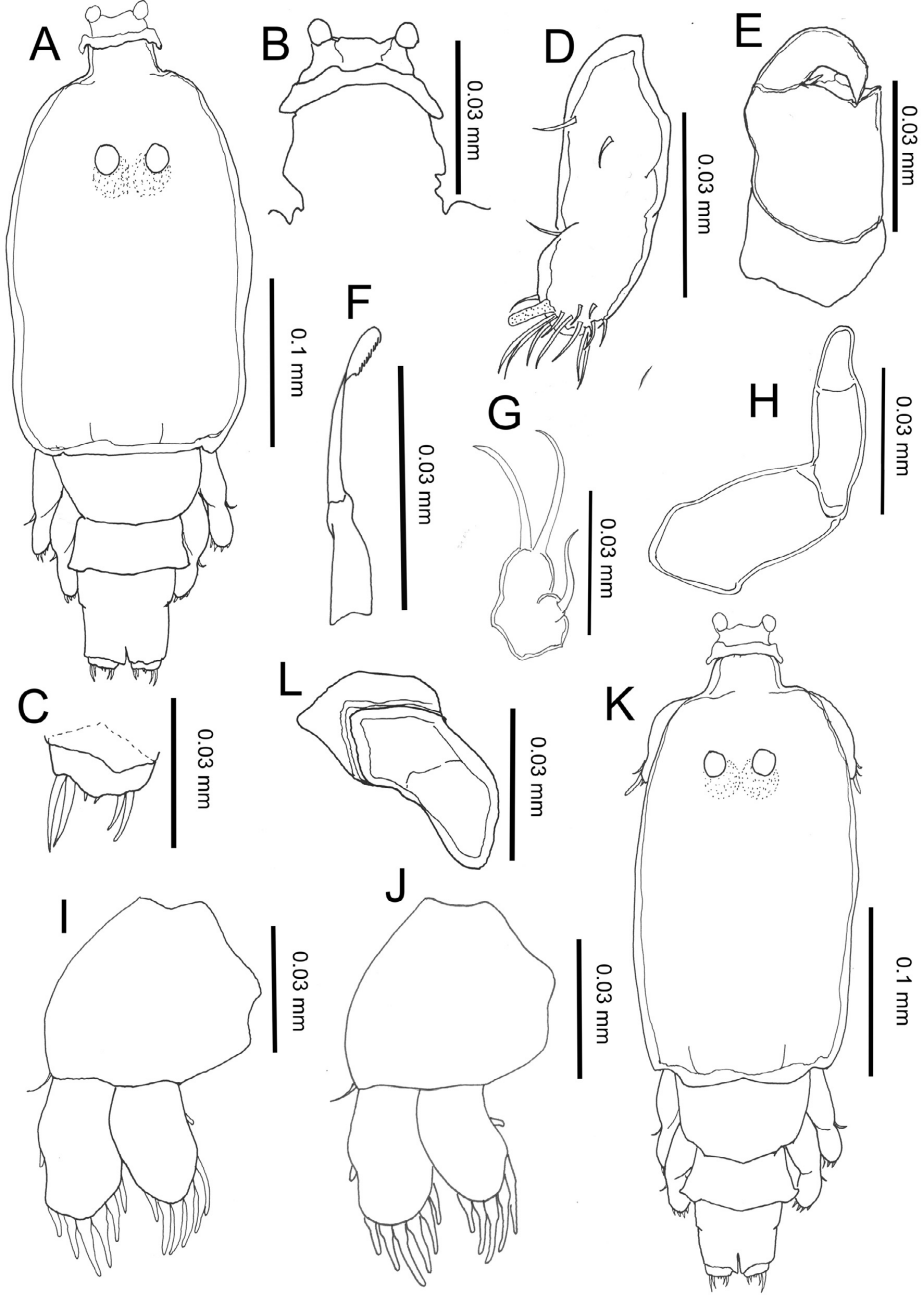
*Peniculus minuticaudae* Shiino, 1956. First chalimus stage: A, female, habitus, dorsal view; B, frontal filament; C, caudal ramus, dorsal view; D, antennule; E, antenna; F, mandible; G, maxillule; H, maxilla; I, leg 1, anterior surface; J, leg 2, anterior surface; K, male, habitus, dorsal view; L, anlagen of maxilliped.

Body (Figure 3A) slightly larger than that of copepodid. Cephalothorax about 1.48 times longer than free post-cephalothoracic somites combined. Frontal filament (Figure 3B) bearing single hood extending from cephalothorax, attached to fin rays by two short strands. Naupliar eyes present. Second pedigerous somite wider than long; third free somite and anal somite indistinctly separated. Anal somite bearing short caudal rami (Figure 3C) armed with 6 naked setae of unequal length.

Antennule (Figure 3D) indistinctly 2-segmented, proximal part bearing three marginal setae, distal part having 13 fine setae and aesthetasc. Antenna (Figure 3E) indistinctly 3-segmented, chelate; proximal segment large; middle segment with two pointed processes medially; distal segment claw-like, with single minute seta basally. Mandible (Figure 3F), maxillule (Figure 3G) and maxilla (Figure 3H) as in copepodid. Mouth cone not developed. Maxilliped absent. Legs 1 (Figure 3I) and 2 (Figure 3J) biramous, comprising protopod with unisegmented rami. Armature formula of legs is shown in Table 1.

##### Remarks

The first chalimus differs from copepodid in general appearance, body shape, the presence of a frontal filament, the possession of finer setae on the antennule, the structure of legs 1–3 and the absence of plumose setae on the caudal rami. Legs 1 and 2 comprised protopod, exopod and endopod but the setae on the rami are simple with no apparent differentiation between spines and setae at this stage. In comparison to other pennellids, differences can be seen in the antenna and the maxilla. In *Cardiodectes* sp. the tips of the antennary claw and the terminal claw of the maxilla are both split into 3 processes [12].

#### First chalimus, male (Figure 3K–L)

Body length (based on four individuals collected from *S. cirrhifer*): 3.27 (3.15–3.36) mm.

Body (Figure 3K) and other features similar to those of female. Maxilliped (Figure 3L) present as anlagen just behind maxilla.

##### Remarks

The presence of the maxilliped anlagen of the first chalimus represents the first appearance of sexual dimorphism in *P. minuticaudae*. The first appearance of sexual dimorphism is also at the first chalimus in *Cardiodectes medusaeus* (Wilson, 1908) [25].

#### Second chalimus, female (Figure 4A–K)

Body length (based on three individuals collected from *S. cirrhifer*): 3.48 (3.23–3.73) mm.

**Figure 4.**
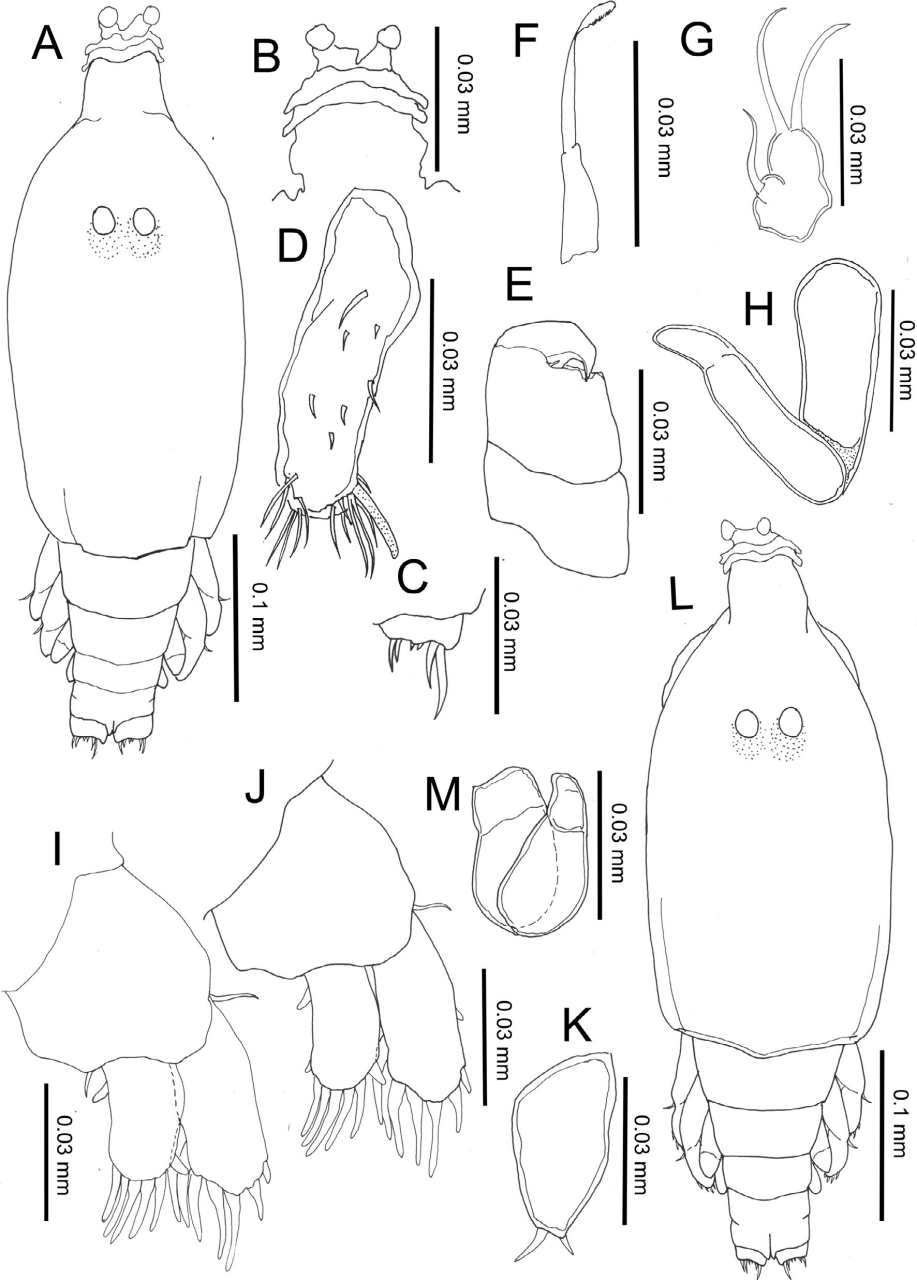
*Peniculus minuticaudae* Shiino, 1956. Second chalimus stage: A, female, habitus, dorsal view; B, frontal filament; C, caudal ramus, dorsal view; D, antennule; E, antenna; F, mandible; G, maxillule; H, maxilla; I, leg 1, anterior surface; J, leg 2, anterior surface; K, leg 3, anterior surface; L, male, habitus, dorsal view; M, maxilliped.

Body (Figure 4A) with elongated cephalothorax and four free somites. Cephalothorax about 1.41 times longer than free post-cephalothoracic somites combined. Frontal filament (Figure 4B) longer than in preceding stage; two remnants present at tip of frontal filament. Naupliar eyes present. Second pedigerous somite wider than long. Fourth pedigerous somite bearing anlagen of leg 4 ventrolaterally. Anal somite wider than long, bearing caudal rami (Figure 4C) with 6 naked setae of unequal length.

Antennule (Figure 4D) indistinctly 2-segmented; proximal part bearing 7 marginal setae; distal part with 13 setae and aesthetasc. Antenna (Figure 4E) similar to that of preceding stage. Mandible (Figure 4F), maxillule (Figure 4G) and maxilla (Figure 4H) similar to those of preceding stage. Maxilliped absent. Legs 1 (Figure 4I) and 2 (Figure 4J) biramous, comprising protopod with unisegmented rami. Leg 3 (Figure 4K) uniramous with 2 setae at tip. Armature of legs given in Table 1.

##### Remarks

The second chalimus differs from the preceding stage in the frontal filament and the setation on legs. The frontal filament is quite prominent and more elongate in comparison to that of first chalimus female and two remnants are visible. In leg 1, 1 additional seta is present on the posterior margin of the protopod. In leg 2, 1 and 2 setae are added to the exopod and endopod, respectively. The characteristic features of leg segmentation and setation are similar to those of *L. branchialis* [29] and *L. sprattae* [26]. Leg 3 is represented by a single ramus equipped with 2 simple setae terminally and leg 4 by an anlagen on the fourth thoracic somite. In comparison, leg 3 of the second chalimus of *Cardiodectes* sp. bears 6 setae and the rudimentary protuberance of leg 4 is specific to the female only [12].

#### Second chalimus, male (Figure 4L–M)

Body length (based on four individuals collected from *S. cirrhifer*): 3.45 (3.28–3.80) mm.

Body (Figure 4L) similar to that of female. Cephalothorax about 1.39 times longer than free post-cephalothoracic somites combined. Other features similar to those of female, except for presence of maxilliped (Figure 4M). Maxilliped 2-segmented; proximal segment large and stout; distal segment tapering distally into blunt claw.

##### Remarks

Generally the body and appendages are similar to those of the female except for the presence of the maxilliped and the anal somite, which is slightly longer than in the female.

#### Third chalimus, female (Figure 5A–L)

Body length (based on five individuals collected from *S. cirrhifer*): 4.44 (4.41–4.47) mm.

**Figure 5.**
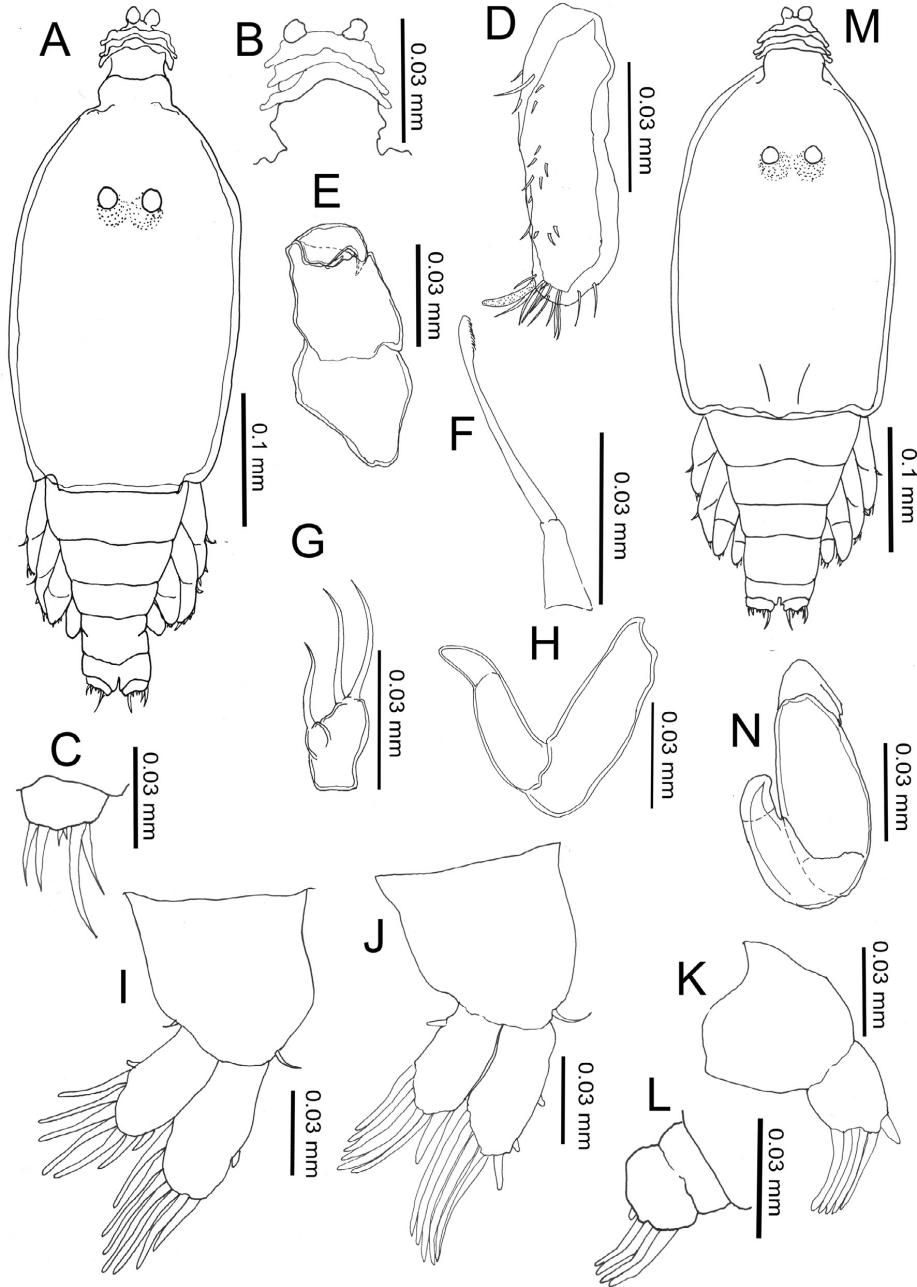
*Peniculus minuticaudae* Shiino, 1956. Third chalimus stage: A, female, habitus, dorsal view; B, frontal filament; C, caudal ramus, dorsal view; D, antennule; E, antenna; F, mandible; G, maxillule; H, maxilla; I, leg 1, anterior surface; J, leg 2, anterior surface; K, leg 3, anterior surface; L, leg 4, anterior surface; M, male, habitus, dorsal view; N, maxilliped.

Body (Figure 5A) slender with cephalothorax about 1.5 times longer than free post-cephalothoracic somites combined, widest at mid-length. Frontal filament (Figure 5B) with three remnants. Anal somite bearing caudal rami (Figure 5C) with 6 setae of unequal length.

Antennule (Figure 5D) indistinctly 2-segmented; proximal part bearing 15 setae along anterior margin; distal part with 13 setae and aesthetasc. Antenna (Figure 5E), as in preceding stage. Mandible (Figure 5F), maxillule (Figure 5G), and maxilla (Figure 5H) as in preceding stage. Legs 1 (Figure 5I) and 2 (Figure 5J) biramous, comprising protopod with unisegmented rami. Legs 3 (Figure 5K) and 4 (Figure 5L), uniramous, 2-segmented. All legs armed with naked setae. Armature of legs given in Table 1.

##### Remarks

The third stage has one additional free somite in comparison to the second stage. Other differences are the additional remnants on the frontal filament, the setation of the antennule, the development of the third and fourth legs and also the setation of all legs.

#### Third chalimus, male (Figure 5M–N)

Body length (based on four individuals collected from *S. cirrhifer*): 3.92 (3.89–3.94) mm.

Body (Figure 5M) stubbier with cephalothorax about 1.51 times longer than free post-cephalothoracic somites combined. Fourth free somite wider than long. All other features similar to those of female except for presence of maxilliped. Maxilliped (Figure 5N) 2-segmented; proximal segment robust, unarmed; terminal segment tapering distally into blunt claw.

##### Remarks

Sexual dimorphism can be seen in the general body appearance, which is stubbier than female, the presence of the maxilliped and the shape of the fourth free somite (which is shorter and wider than that of the female).

#### Fourth chalimus, female (Figure 6A–L)

Body length (based on five individuals collected from *S. cirrhifer*): 4.35 (4.14–4.51) mm.

**Figure 6.**
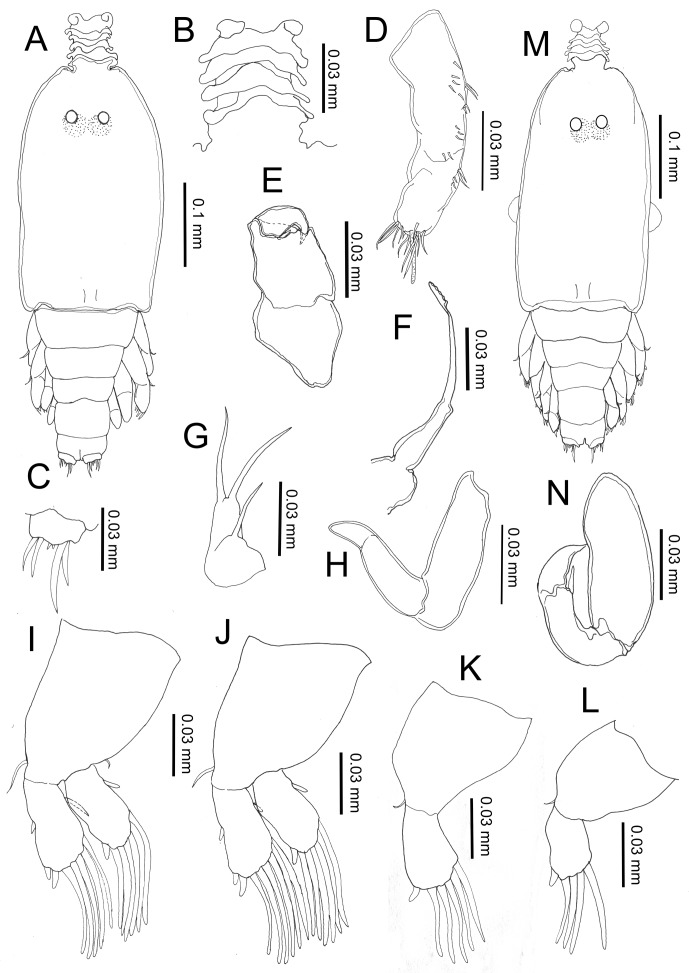
*Peniculus minuticaudae* Shiino, 1956. Fourth chalimus stage: A, female, habitus, dorsal view; B, frontal filament; C, caudal ramus, dorsal view; D, antennule; E, antenna; F, mandible; G, maxillule; H, maxilla; I, leg 1, anterior surface; J, leg 2, anterior surface; K, leg 3, anterior; L, leg 4, anterior surface; M, male, habitus, dorsal view; N, maxilliped.

Body (Figure 6A) with more distinct body segmentation. Cephalothorax about 1.5 times longer than free post-cephalothoracic somites combined. Frontal filament (Figure 6B) with four remnants. Nauplius eyes conspicuous. Caudal rami (Figure 6C) as in preceding stage.

Antennule (Figure 6D) indistinctly 2-segmented, proximal part bearing 18 setae along anterior margin, distal part bearing 13 setae and aesthetasc. Antenna (Figure 6E) as in preceding stage. Mandible (Figure 6F), maxillule (Figure 6G) and maxilla (Figure 6H) as in preceding stage. Legs 1 (Figure 6I) and 2 (Figure 6J) biramous, each composed of protopod and 1-segmented rami. Legs 3 (Figure 6K) and 4 (Figure 6L) uniramous, 2-segmented. Armature of legs given in Table 1.

##### Remarks

This stage is easily distinguished from the preceding stage by: almost all appendages have characteristics close to the adult form; the four remnants on the frontal filament are clearly visible; all legs have the adult number of setal elements, the exopod and endopod are elongated with setae protruded from some indentation points, which in adults are separated into two segments. The fourth chalimus female in the present study is similar to the late chalimus female the previous description [24], except for the setation on legs 1 and 2 and the teeth count on mandible.

#### Fourth chalimus, male (Figure 6M–N)

Body length (based on two individuals collected from *S. cirrhifer*): 4.31 (4.13–4.50) mm.

Body (Figure 6M) shorter than that of female. Cephalothorax longer than wide, about 1.5 times longer than free post-cephalothoracic somites combined. Appendages similar to those of female except for presence of maxilliped. Maxilliped (Figure 6N) 2-segmented; proximal segment robust, unarmed; terminal segment tapering distally into pointed claw having single element midway along concave margin.

##### Remarks

The general body length is shorter than in the female and the strong maxilliped of the male represents distinct sexual dimorphism. The body segmentation and form of the maxilliped are similar to those of the late chalimus male of previous description [24]. However, the setation of legs 1 and 2 of the fourth chalimus male in the present study differs from that described of the previous description [24] of the late chalimus male.

#### Pre-metamorphic adult female (Figure 7A–L)

Body length (based on six individuals collected from *S. cirrhifer*): 5.90 (5.60–6.60) mm.

**Figure 7.**
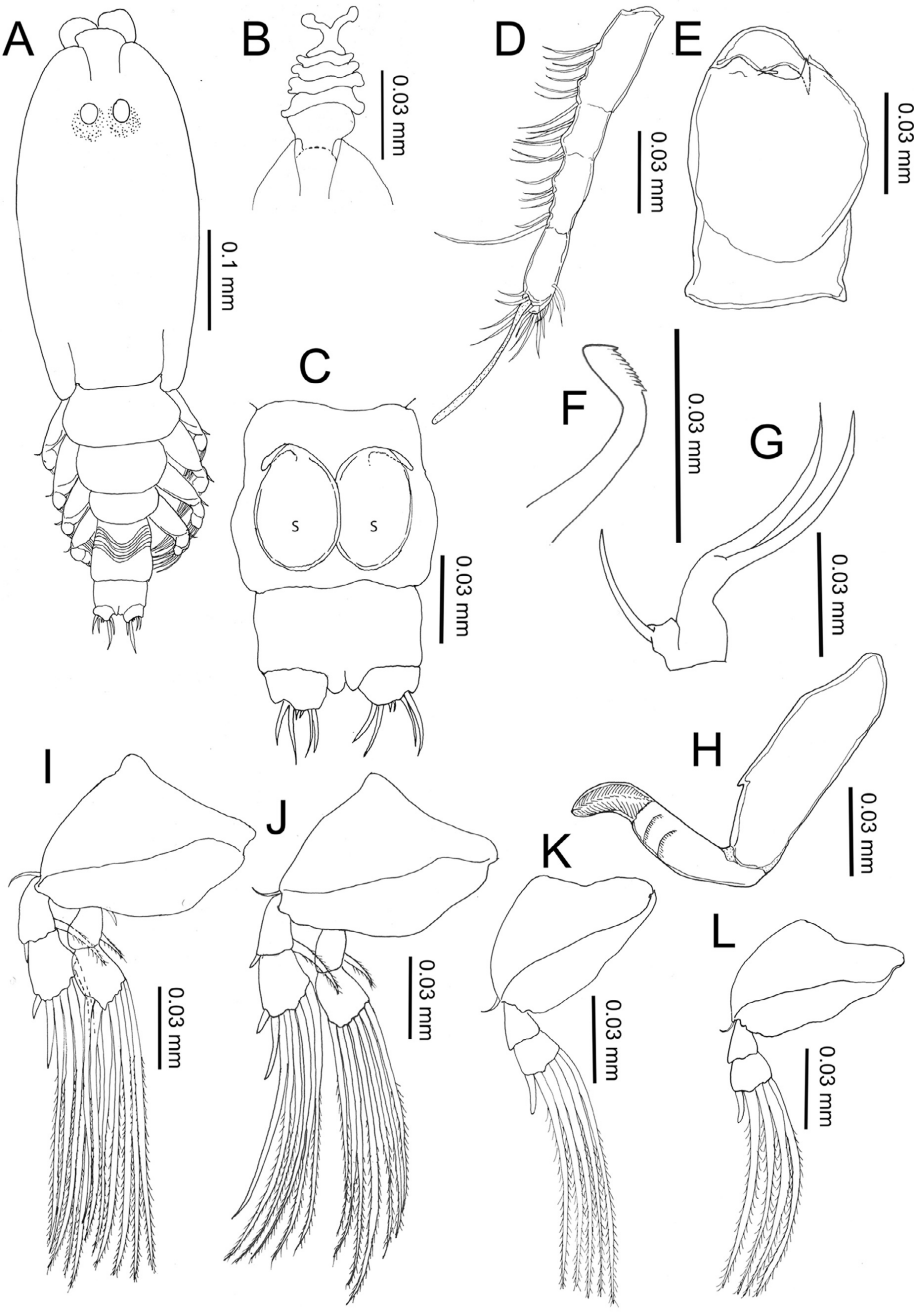
*Peniculus minuticaudae* Shiino, 1956. Pre-metamorphic adult female: A, habitus, dorsal view; B, frontal filament; C, posterior region with attached spermatophores, ventral view, s = spermatophore. D, antennule; E, antenna; F, mandible; G, maxillule; H, maxilla; I, leg 1, anterior; J, leg 2, anterior; K, leg 3, anterior; L, leg 4, anterior.

Body (Figure 7A) slender with distinct five post-cephalothoracic somites. Cephalothorax with large, conical rostrum, longer than wide, about 1.5 times longer than free post-cephalothoracic somites combined. Temporary frontal filament with five remnants (Figure 7B). Nauplius eyes conspicuous. Genital complex long, with surface showing transverse striations. Abdomen short, wider than long; carrying caudal rami with 6 setae of unequal length (Figure 7C).

Antennule (Figure 7D) 4-segmented, with armature formula of 7, 6, 8, 13 + ae. Antenna (Figure 7E) indistinctly 3-segmented, chelate; proximal segment large, middle segment robust, bearing 2 pointed processes on inner margin; distal segment claw-like with minute seta at base. Oral cone well developed, located midventrally on surface of cephalothorax. Mandible (Figure 7F) rod-like with 10 teeth and pointed tip. Maxillule (Figure 7G) bilobed with 1 and 2 setae at tip, respectively. Maxilla (Figure 7H) 2-segmented; proximal segment with single process anteriorly; distal segment with transverse striations and two rows of fine setulose ornamentations. Legs 1 (Figure 7I) and 2 (Figure 7J) with coxa, basis, and 2-segmented rami. Legs 3 (Figure 7K) and 4 (Figure 7L) with coxa, basis and 2-segmented exopod only. All rami armed with plumose setae. Armature of legs given in Table 1.

##### Remarks

The adult male of *P. minuticaudae* was first described by Okawachi et al. [24]. Sexual dimorphism between adult male and pre-metamorphic adult female can be seen in the body, the antenna and in the genital structures. The body of the male is composed of seven post-cephalothoracic somites while the pre-metamorphic adult female has only five post-cephalothoracic somites. The antenna of the male is similar to that of the chalimus stages while, in the female, it is swollen proximally. The post-metamorphic adult females show huge morphological differences from the pre-metamorphic stage. The body segmentation of post-metamorphic adult female is reduced due to the incorporation of fourth pedigerous somite into the expanded genital complex to form the trunk region [24, 27, 30]. The abdominal somite has also become indistinctly separated from the trunk [24, 27, 30]. The caudal rami that were located at posterior end of pre-metamorphic female have been pushed towards the posteroventral part of the post-metamorphic female. The post-metamorphic females also lack antennules and the rami on the legs [24, 27, 30], which are retained in the pre-metamorphic female, for swimming purposes.

## Discussion

### Complete life cycle of *P. minuticaudae*

**Figure 8.**
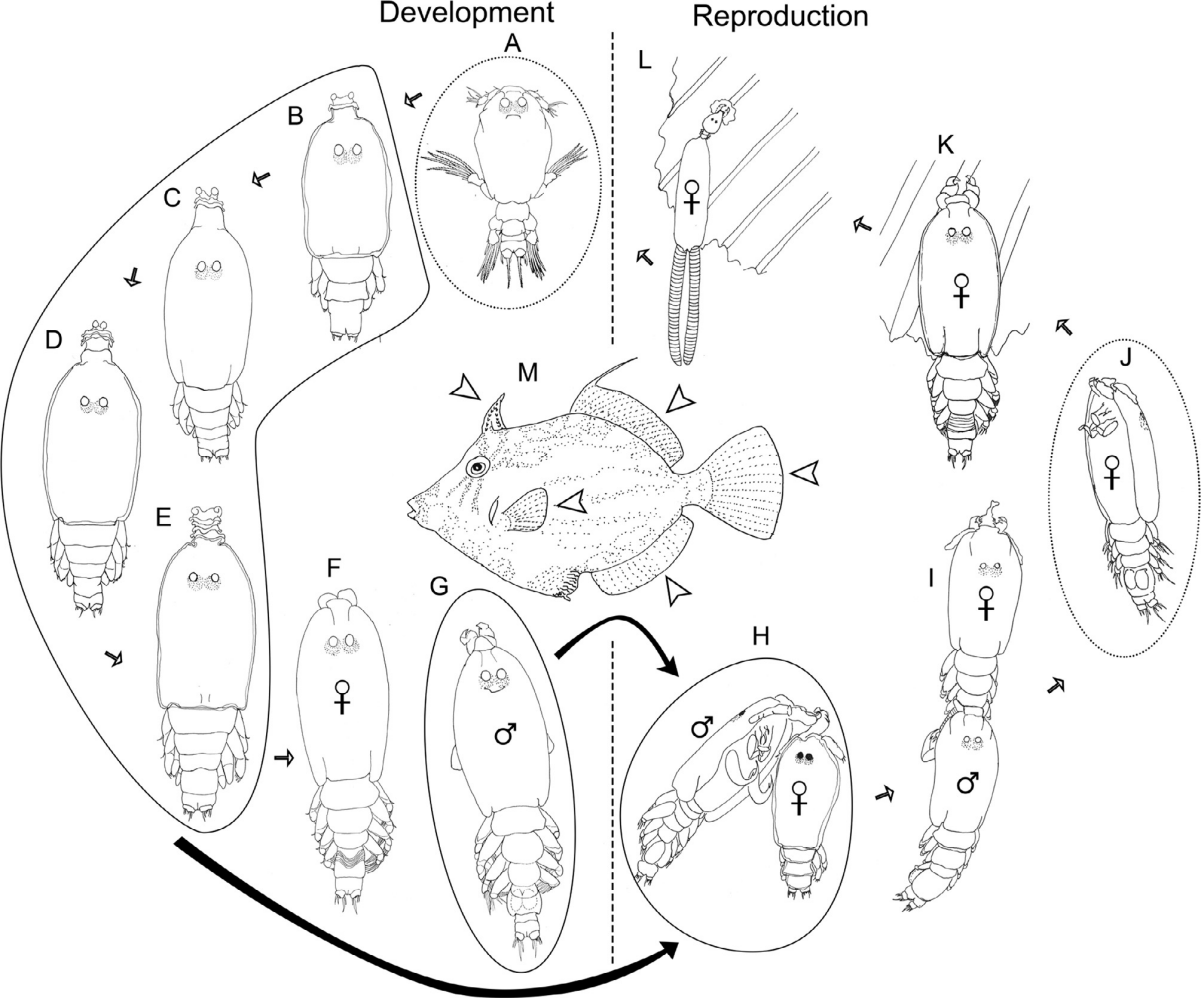
Schematic life cycle of *Peniculus minuticaudae* Shiino, 1956. A, infective copepodid; B, chalimus I; C, chalimus II; D, chalimus III; E, chalimus IV; F, pre-metamorphic adult female; G, adult male; H, pre-copulation guarding of chalimus I female by adult male; I, copulation of adult male to pre-metamorphic adult female; J, fertilized pre-metamorphic adult female with spermatophores, detached from temporary frontal filament and swimming to locate new settlement site; K, fertilized pre-metamorphic adult female clinging to the new settlement site on the fin ray of host; L, ovigerous post-metamorphic female on the fin of fish host; M, fish host, threadsail filefish (*Stephanolepis cirrhifer*). Arrowheads show infection sites of *P. minuticaudae* on the host. Stages involved in precopulation surrounded by continuous lines; free-swimming stages surrounded by dotted lines.

**Figure 9.**
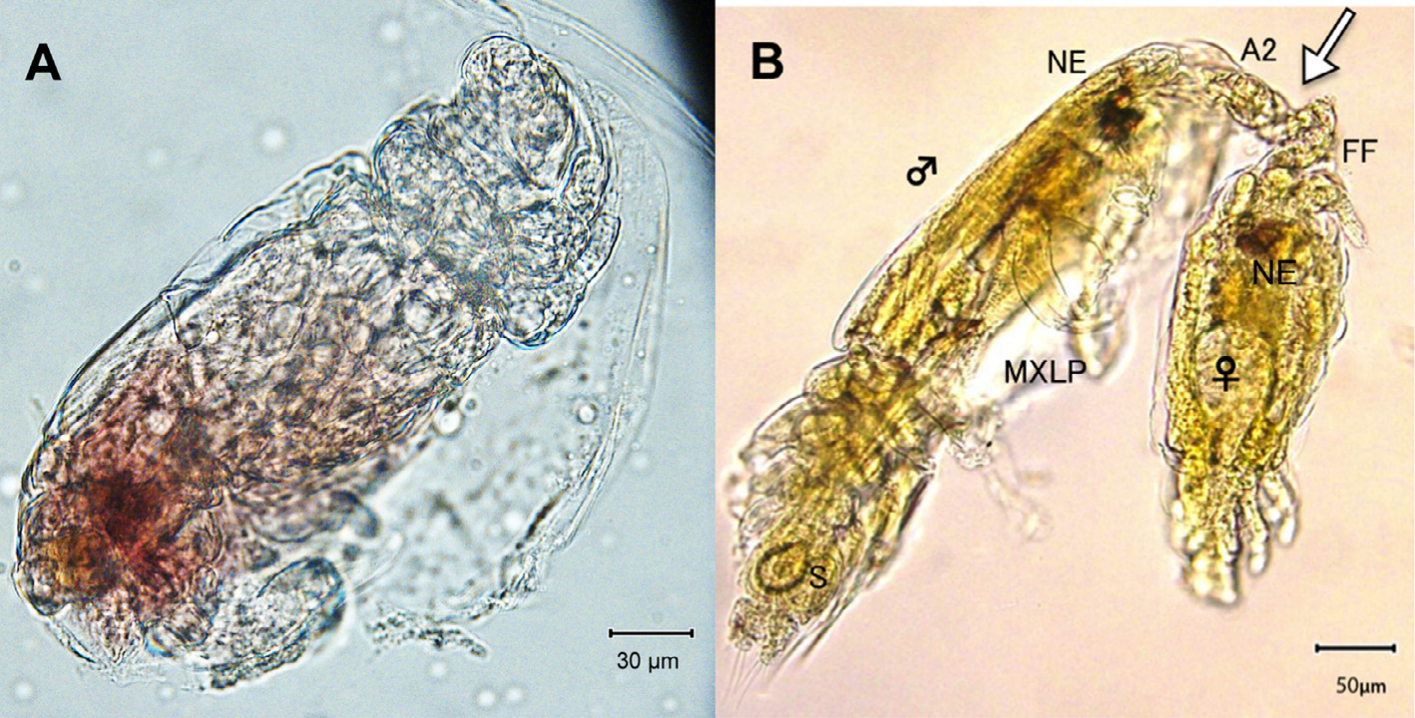
*Peniculus minuticaudae* Shiino, 1956. A, infective copepodid within the egg membrane; B, pre-copulation guarding of chalimus I female by adult male. Arrow showing the male’s antennae which grasp the female’s frontal filament. NE = nauplius eyes, MXLP = maxilliped, S = spermatophore, A2 = antennae, FF = frontal filament.

The presumed complete life cycle of *P. minuticaudae* based on the discovery of all stages from a single host *S. cirrhifer* (Figure 8) is the first to be elucidated for the genus *Peniculus*. Overall, the life cycle of *P. minuticaudae* consists of six developmental stages separated by five moults, the infective copepodid (Figure 8A), four chalimi (Figure 8B–E) and adult (Figure 8F, G). Through observation of hatching of the egg strings incubated under laboratory conditions, we could confirm that the hatching stage of *P. minuticaudae* is the copepodid (Figure 9A). The hatched infective copepodid actively swims and locates a host [6]. After settlement on the host, particularly on the fins, the copepodid moults into the first chalimus stage. *Peniculus minuticaudae* has four chalimus stages prior to the final moult to adult. The presence of complete and well-developed swimming legs in the pre-metamorphic adult female and adult male suggests that they have ability to detach from the host for copulation, or to search for another suitable host or site of final settlement.

Copulation in *P. minuticaudae* (Figure 8I) is likely to be similar to that in other pennellids, as described by Ho [12] and Schram [26]. Soon after copulation, the fertilized pre-metamorphic female detaches and swims to find a new settlement site (Figure 8J). After final settlement (Figure 8K), the pre-metamorphic adult female undergoes massive differential growth, develops into the post-metamorphic adult female (Figure 8L), and begins to produce eggs. Precopulatory mate guarding (Figure 8H) was also observed between adult male and various developmental stages of the female, including the first chalimus stage female (Figure 9B). The male grasps the female at the base of its frontal filament. Such precopulatory behavior has also been reported in *L. branchialis* [2, 5].

In the present study, all stages of *P. minuticaudae* from copepodid to post-metamorphic adult female were found infecting the fins of *S. cirrhifer*. However, in the case of individual fish burdened with a high density of parasites, some of the pennellids can also be found attached to the skin near the fins. All stages of *P. minuticaudae* attached to the host by grasping the fin tissues using their antennae, except for chalimus stages, which attach by means of their frontal filament. Unlike *L. branchialis* [8] and *L. sprattae* [26], no copepodid of *P. minuticaudae* was found with a frontal filament, which suggests that the extrusion of the frontal filament might occur very shortly before the moult to first chalimus stage. However, two adult females were found attached to the fin ray of a host by means of temporary frontal filament [6, 23, 31] as also observed by Ho [12] in *Cardiodectes* sp. specimens. Attachment of the post-metamorphic adult females was made more secure because the cephalothorax was encapsulated within hyperplastic epithelial tissue of the fin, that presumably developed in response to the feeding activity, or presence, of the copepod.

Okawachi et al. [24] concluded that the life cycle of pennellids can be divided into four phases, i.e., first free-living, first sessile (or chalimus) phase, second free-living and second sessile phase. Two swimming stages i.e., the infective copepodid and the fertilized pre-metamorphic female determine the settlement site for the first and second sessile phases, respectively [25, 26, 29]. These two stages of *P. minuticaudae* were found to infect a single host, on the same site particularly the fins, together with all other stages. From our new findings we confirm the suggestion of Okawachi et al. [24] that *P. minuticaudae* can complete its life cycle on a single host.

### Comparison of life cycle among pennellids

The complete life cycle of pennellids has so far been described only for three genera and species: *L. branchialis* [8, 29], *C. medusaeus* [12, 25] and *L. sprattae* [26] and now the fourth species *P. minuticaudae*. The present study on *P. minuticaudae* sheds new light into the life cycle of pennellids. The characteristics including the life cycle of all four genera (Pennellidae) are compared in this study (Table 2).

**Table 2.** Comparison on the life cycle and characteristics of four pennellids (*Lernaeocera branchialis, Cardiodectes medusaeus, Lernaeenicus sprattae* and *Peniculus minuticaudae*).

Pennellids	*Lernaeocera branchialis* (Linnaeus, 1767)	*Cardiodectes medusaeus* (Wilson, 1908)	*Lernaeenicus sprattae* (Sowerby, 1806)	*Peniculus minuticaudae* (Shiino, 1956)
Characteristics				
Developmental stages	8 (2 naupliar, 1 copepodid, 4 chalimi, adult)	5 (1 copepodid, 3 chalimi, adult)	8 (2 naupliar, 1 copepodid, 4 chalimi, adult)	6 (1 copepodid, 4 chalimi, adult)
Host(s) needed to complete life cycle	Double	Double	Single	Single
Intermediate host	Mainly fishes, Pleuronectidae	Pelagic gastropods, mainly Cavolinidae and Janthinidae	–	–
Definitive host	Fish, Gadidae	Fish, Myctophidae	Fish, Clupeidae	Fishes, Monacanthidae, Chaetodontidae
Infection site of copepodid stage	Gill lamellae	Gill lamellae/mantle tissues	Body surface and fins	Fins
Infection site of post-metamorphic female	Burrowing through the gill arch to reach the heart of the fish host	Burrowing from various parts of the ventral surface of the fish host to reach the heart	Eyes	Fins
Possible food source of larval stages and post-metamorphic female	Blood	Blood	Blood, coelomic and tissues fluid	Presumably epithelium tissue and mucous
Range size of post-metamorphic female	20–50 mm	8.5–15 mm	12–18 mm	5–6 mm
References	Sproston [29], Brooker et al. [7, 8], Kearn [19]	Ho [12], Perkins [25]	Schram [26], Kearn [19]	Shiino [27], Nagasawa et al. [20], Venmathi Maran et al. [30], Okawachi et al. [24], present study

The basic life cycle of copepods comprises two phases with six naupliar stages and five post-naupliar stages prior to the adult stage [6]. However, naupliar phase abbreviation is a common phenomenon for siphonostomatoid copepods and the brief summary of abbreviation of the naupliar phase among siphonostomatoid copepods was given by Izawa [16]. Some siphonostomatoids of the families Lernaeopodidae H. Milne-Edwards, 1840 [17], Nicothoidae Dana, 1852 [21, 22] and Pennellidae [12, 15, 25] showed the most abbreviated naupliar phase by skipping all the stages and hatching directly as the infective copepodid [6, 16]. While abbreviation of the naupliar phase is common, siphonostomatoid copepods retain the basic five post-naupliar stages prior to adult [6, 23, 31]. However, due to the transition from a free-living to a parasitic mode of life, after the settlement of infective copepodid on the host, most siphonostomatoid copepodids parasitizing fishes undergo copepodid form modification by attaching to the host by means of a frontal filament and these forms are referred to as chalimus larvae [11, 6, 23, 31].

Among pennellids (Table 2), *L. branchialis* and *L. sprattae* retain the naupliar phase and have a total of seven developmental stages prior to the adult (two naupliar, one copepodid, four chalimus). In contrast, *C. medusaeus* and *P. minuticaudae* show naupliar phase abbreviation and hatch directly as the infective copepodid. *Peniculus minuticaudae* shares the similarity in the pattern of post-naupliar stages with other two genera, *L. branchialis* and *L. sprattae,* by having one copepodid and four chalimus stages prior to adult. However, *C. medusaeus* was reported with lacking one chalimus stage in comparison to other pennellids (cf. Table 2) [12, 25]. Since abbreviation of post-naupliar stages is not common among siphonostomatoid copepods, revision of the life cycle of *C. medusaeus* might be necessary to confirm the unusual feature.

The involvement of intermediate and definitive hosts in pennellid life cycles varies (cf. Table 2): *Lernaeocera branchialis* and *C. medusaeus* require two hosts [12, 25, 29], while *L. sprattae* [1, 26] and *P. minuticaudae* (present study) are able to complete their life cycle on a single host. For *P. minuticaudae*, our observations showed that all developmental stages infected at the same site, particularly the fins. In the case of *L. sprattae*, the infection site of the adult female after copulation differs from that of the infective copepodid and chalimus stages. The adult female particularly infects the eyes of the fish host, while other developmental stages infect its fins and body surface [1, 26].

The body size of the post-metamorphic adult female of *P. minuticaudae* is the smallest of the pennellids compared in Table 2. Among pennellids, *Peniculus*, *Peniculisa*, *Exopenna* Boxshall, 1986, and *Parinia* Kazachenko & Avdeev, 1977 are categorized as ectoparasites, while the rest are mesoparasites [4, 18]. Judging from the method of attachment, it is suggested that post-metamorphic adult female of *P. minuticaudae* might ingest epithelium and mucus from the fin, in contrast to other pennellids, which are known as blood-feeders [7, 19, 25]. The feeding type might be a factor influencing size differences among pennellids.

The pathogenicity of *P. minuticaudae* has not yet been studied in detail. However, the findings of high prevalence and intensity on cultured fishes [10, 20, 30] and the mortality of aquarium-kept fishes [24] showed that *P. minuticaudae* could be a potential pest, harming fishes kept in captivity [20, 24, 30].
